# Impact of medical technologies may be predicted using constructed graph bibliometrics

**DOI:** 10.1038/s41598-024-52233-x

**Published:** 2024-01-29

**Authors:** Lawrence Jiang, Ashir Raza, Abdel-Badih El Ariss, David Chen, Nicole Danaher-Garcia, Jarone Lee, Shuhan He

**Affiliations:** 1 Duke University, Durham, USA; 2 Department of Emergency Medicine, Massachusetts General Hospital, Boston, USA; 3 Temerty Faculty of Medicine, University of Toronto, Toronto, Canada; 4 School of Healthcare Leadership, Institute of Health Professions, Boston, USA; 5 Trauma, Emergency Surgery, Surgical Critical Care, Massachusetts General Hospital, Boston, USA; 6 Lab of Computer Science, Massachusetts General Hospital, Boston, USA

**Keywords:** Medical research, Computational science

## Abstract

Scientific research is driven by allocation of funding to different research projects based in part on the predicted scientific impact of the work. Data-driven algorithms can inform decision-making of scarce funding resources by identifying likely high-impact studies using bibliometrics. Compared to standardized citation-based metrics alone, we utilize a machine learning pipeline that analyzes high-dimensional relationships among a range of bibliometric features to improve the accuracy of predicting high-impact research. Random forest classification models were trained using 28 bibliometric features calculated from a dataset of 1,485,958 publications in medicine to retrospectively predict whether a publication would become high-impact. For each random forest model, the balanced accuracy score was above 0.95 and the area under the receiver operating characteristic curve was above 0.99. The high performance of high impact research prediction using our proposed models show that machine learning technologies are promising algorithms that can support funding decision-making for medical research.

## Introduction

Scientific research is a driving force of innovation designed to expand the frontiers of human knowledge and improve economic and social progress. However, research policy and exploration of promising research directions are shaped in part by the decisions of funding bodies, such as governments and universities, as well as for-profit and nonprofit private entities, to fund these research studies^1,2^. Determining which proposed research projects are funded based on impact remains a dynamic process that involves a combination of peer review and quantitative research metrics^2,3^. The funding decision process requires transparency in the way public research funds are allocated based on peer review and metrics. Transparency is needed to ensure reproducibility through increased use of publicly available data for responsible decision-making^4,5^.

Standardized citation metrics of research articles in a scientific field and scientist profiles may be used in part to inform the decision-making to fund new scientific projects^6–8^. Using the number of citations alone as the sole quality indicator of research is limited due to its narrow scope that only measures the uptake of the work by other researchers^9,10^. Moreover, citations are lagging indicators of research impact that vary widely by journal and scientific field^11^. The limitations of relying solely on standardized citation metrics in the funding decision process can have a significant impact on the prospective development of science. Funding bodies may overlook potentially impactful research projects that are not immediately recognized by the scientific community. Additionally, researchers may prioritize producing work that is more likely to be cited, rather than pursuing research that is more innovative or impactful, which can slow scientific progress^12^.

Recent research to extract signals from network science-enabled knowledge graphs has been used to quantify domain knowledge in health and life sciences^13^, materials science^14^, and drug discovery^15^. Recent work by Weis and Jacobson^16^ demonstrated the promising performance of leveraging knowledge graph dynamics and machine learning algorithms to a biomedical-focused dataset to identify innovative research of likely future importance. Additionally, machine learning algorithms have been used to identify seminal research of likely future significance in drug discovery. To evaluate the performance and reproducability of using machine learning-based classification of high-impact research studies, we designed supervised random forest models trained on graph bibliometrics to predict high-impact research studies.

## Methods

**Figure 1:**
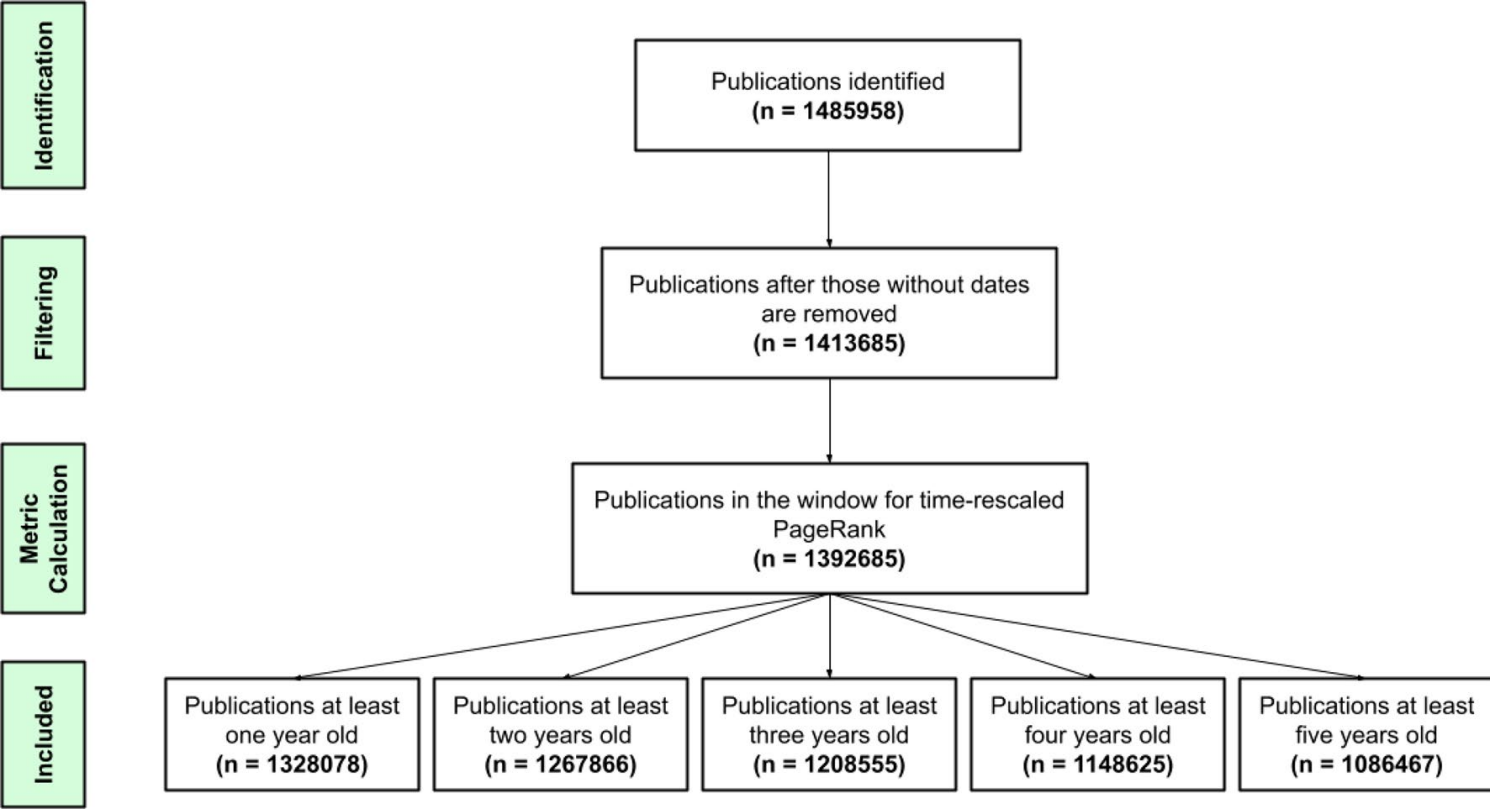
Eligibility of publications. On the date the data were pulled, there were 1,485,958 publications in the 40 journals between 1980 and 2020. Of those, we did not have sufficient information on the date of publication for 72,273 articles. In calculating time-rescaled PageRank, 21,000 randomly selected publications were placed outside the sliding window.

We collected metadata on 1,485,958 publications with non-null author, title, and ISSN data from 40 high-impact medical journals, listed in Table 1, between 1980 and 2020^17^. Data was collected from the APIs of Lens Lab and Elsevier. Since the data was collected in 2021, 2020 was the last year with complete data at the time. Articles without sufficient data on the date they were published were removed. A schematic on the search process of publications included in this study is shown in Fig. 1.

**Table 1:** Journals in dataset.

The New England Journal of Medicine	The Lancet
JAMA—the Journal of the American Medical Association	The BMJ—the British Medical Journal
Annals of Internal Medicine	JAMA Internal Medicine
PLOS Medicine	Lancet Oncology
World Psychiatry	Lancet Neurology
Journal of Clinical Oncology	European Heart Journal
JACC—Journal of the American College of Cardiology	Lancet Infectious Diseases
Lancet Diabetes and Endocrinology	Circulation
Lancet Respiratory Medicine	Gastroenterology
Gut	JAMA Oncology
European Urology	JAMA Psychiatry
American Journal of Psychiatry	Circulation Research
Hepatology	American Journal of Respiratory and Critical Care Medicine
Blood	Journal of Allergy and Clinical Immunology
Annals of the Rheumatic Diseases	JNCI—Journal of the National Cancer Institute
Journal of Hepatology	Intensive Care Medicine
Diabetes Care	Annals of Oncology
Leukaemia	Lancet Psychiatry
European Respiratory Journal	Brain
JAMA Pediatrics	JAMA Neurology

**Figure 2:**
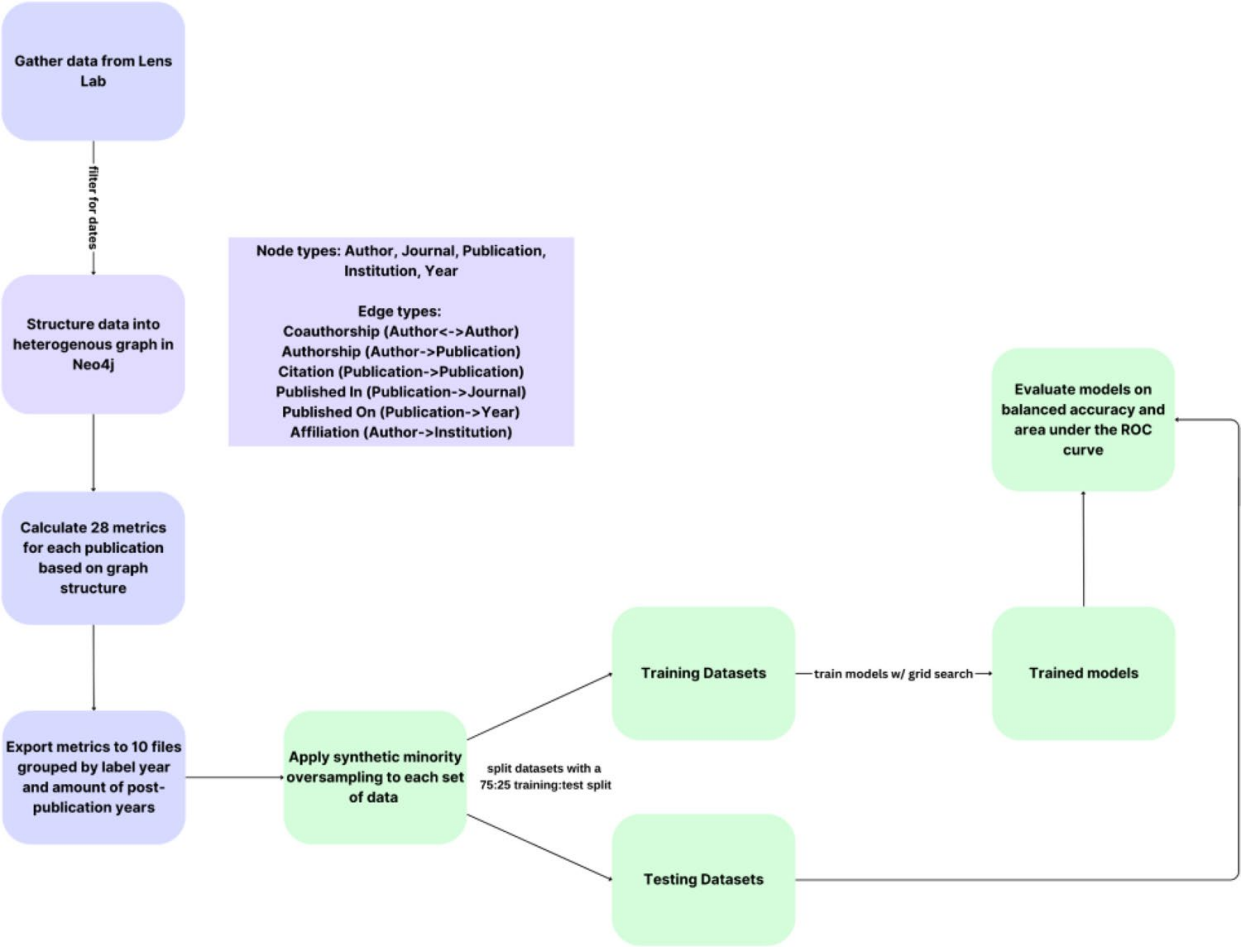
Workflow schematic.

We used Neo4j database and Weis and Jacobson methodology to create nodes and edges for each publication, author, and institution^16^. From the processed database, we generated the same 28 bibliometrics shown in Table 1 of the Weis and Jacobson methodology for each article in our training dataset. The bibliometrics are also available as Table 2 of this article. These constructed graph bibliometrics serve as input features for the proposed machine learning based classifier of high-impact publications. A diagram of the general workflow of the data pre-processing pipeline is shown in Fig. 2.

**Table 2:** Input features.

Variable	Description
Citations per paper	Mean number of citations per paper for papers the author has published
Δ Citations per paper	Change in the mean number of citations per paper for the author over the preceding 2 years
Citations per year	Average number of citations per year for papers the author has published
Maximum citations	Maximum number of citations a paper has received out of all the papers the author has currently published
Rank citations per year	Rank of the author among all other authors in terms of mean citations per year
Total citations	Number of citations author has received
Δ Total citations	Change in the total number of citations for the author over the preceding 2 years
Total papers	Total number of papers published by the author
Δ Total papers	Change in the total number of papers over the preceding 2 years
Citations	Citations collected in the current year
Adopters	Number of unique citing authors in the current year
Author age	Number of years since the year of publication of the author’s first paper
h-index	Author’s h-index
Δ h-index	Change in the author’s h-index over the past 2 years
Recent coauthors	Number of coauthors the author has had in the current and immediately preceding year
Δ Mean journal citations per paper	Two-year change in the mean number of citations per paper of the journals the author has published in
Mean journal citations per paper	Mean number of citations per paper for the journals the author has published in
Δ Mean journal h-index	Two-year change in the mean h-index for the journals the author has published in
Mean journal h-index	Mean h-index for the journals the author has published in
Mean journal maximum citations	Mean of the maximum number of citations any paper published in a journal has received for each journal the author has published in
Mean journal rank citations per paper	Rank of journal in which the author has published, as determined by the mean number of citations per paper
Mean Δ journal total papers	Change in the mean of the total number of papers published in journals the author has published in
Total journals	Total number of journals published in by the author
Mean journal total papers	Mean of the total number of papers published in journals the author has published in
Learned network embedding	Unsupervised embedding of local network structure calculated via application of the node2vec algorithm on the citation graph
Time-rescaled node centrality	Time-balanced network centrality calculated using the full citation network
Unweighted PageRank	PageRank score of author, calculated on the unweighted coauthorship network
Weighted PageRank	PageRank score of author, calculated on the weighted coauthorship network

Adapted from Ref.^16^.

We calculated the Node2vec, a vector representation for nodes based on random walks, for each author node using Neo4j's built-in function. We labeled publications in the top 5% of one feature, time-rescaled PageRank, as high-impact. As a node centrality metric that accounts for temporal bias, time-rescaled PageRank allows for distinction of high-impact articles controlling for their publication dates^28^. To calculate the time-rescaled PageRank metric, we used a sliding average and standard deviation in a sliding window to rescale each publication temporally. In the setup of the sliding window, we placed 21,000 randomly selected publications outside the window.

Using the Python package scikit-learn 1.1.2, we trained random forest supervised classification models with different amounts of post-publication data. We applied imbalanced-learn's synthetic minority oversampling (SMOTE) function to the data using default parameters to account for the imbalanced nature of high and low-impact publications. In total, we trained ten different supervised random forst models. Six models were trained to predict high-impact publications five years after publication and four models were trained to predict high-impact publications using post-publication data from the year of publication. We used a hyperparameter grid search with a tenfold cross-validation procedure to identify the optimal hyperparameter values based on guidance from Van Rijn and Hutter for max features, min samples leaf, and split criterion^36^. After applying SMOTE, we split the data into training and testing sets with a test size proportion of 0.25. Then, we evaluated models on the test set with balanced accuracy (BA) and area under the receiver operating characteristic curve (ROC AUC). We additionally evaluated variable importance with a mean decrease in impurity test on the 0-year data, 5-year label model.

## Results

We evaluated random forest models on the test split with BA and ROC AUC scores. Overall, the ROC AUC and BA scores for each model was above 0.99 and 0.95 respectively (Table 3).

**Table 3:** Outcomes.

Post-publication years	Label year	Total	True positives	True negatives	False positives	False negatives	Balanced accuracy	Receiver operator characteristic
0	5	516,072	252,452	240,696	17,149	5775	0.955564	0.991447
1	5	516,072	254,183	245,824	12,021	4044	0.968859	0.995998
2	5	516,072	255,667	249,337	8508	2560	0.978545	0.997989
3	5	516,072	256,540	252,953	4892	1687	0.987247	0.99173
4	5	516,072	257,257	255,434	2411	970	0.993447	0.999731
5	5	516,072	258,227	257,840	5	0	0.99999	1.0
1	1	617,046	302,923	297,771	10,808	5544	0.973501	0.997095
2	2	594,279	293,639	291,028	6308	3304	0.983829	0.998922
3	3	569,569	282,885	281,004	3921	1759	0.990029	0.999659
4	4	543,605	270,620	270,425	1580	980	0.995292	0.999899

**Figure 3:**
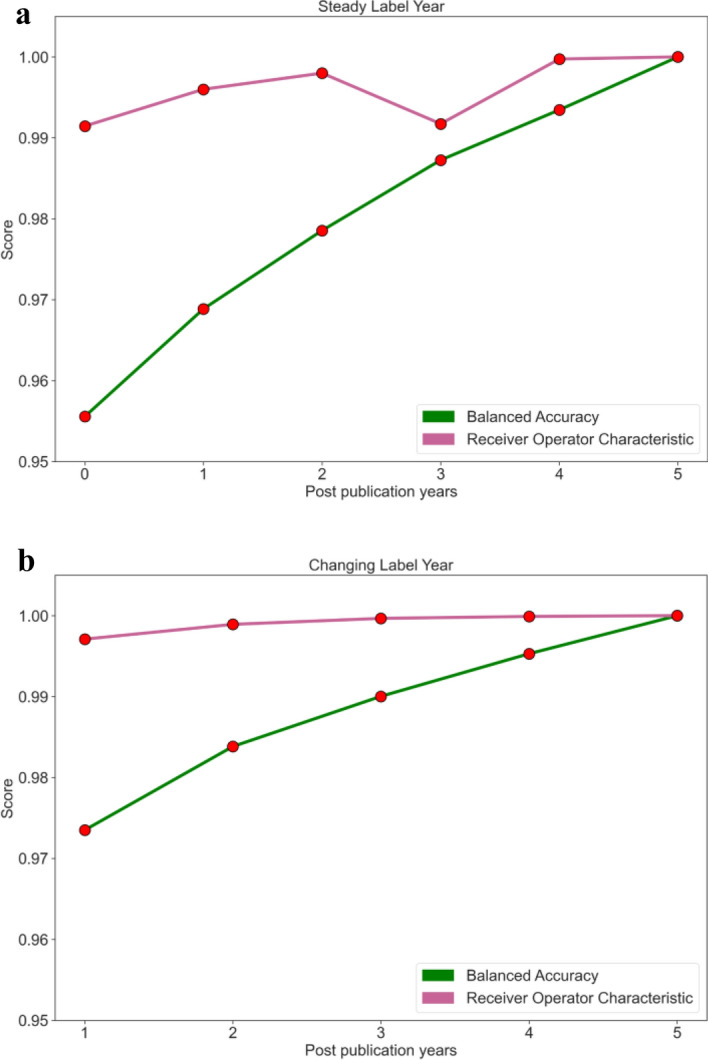
Performance metrics by models. Graphs of BA and ROC AUC for trained models. In (**a**), the data have a label 5 years after publication. In (**b**), the data have a label the same year as the amount of post-publication data made available to the model. (**a**) Corresponds to the models in rows 1–6 of Table 3. (**b**) Corresponds to the models in rows 6–10 of Table 3.

Figure 3 showed that the models generally had higher BA and ROC AUC scores relative to the number of post-publication years. We note that there was a drop in ROC AUC in the 3-year post-publication/5-year label model, suggesting lower performance of the model at the three-year mark.

**Figure 4:**
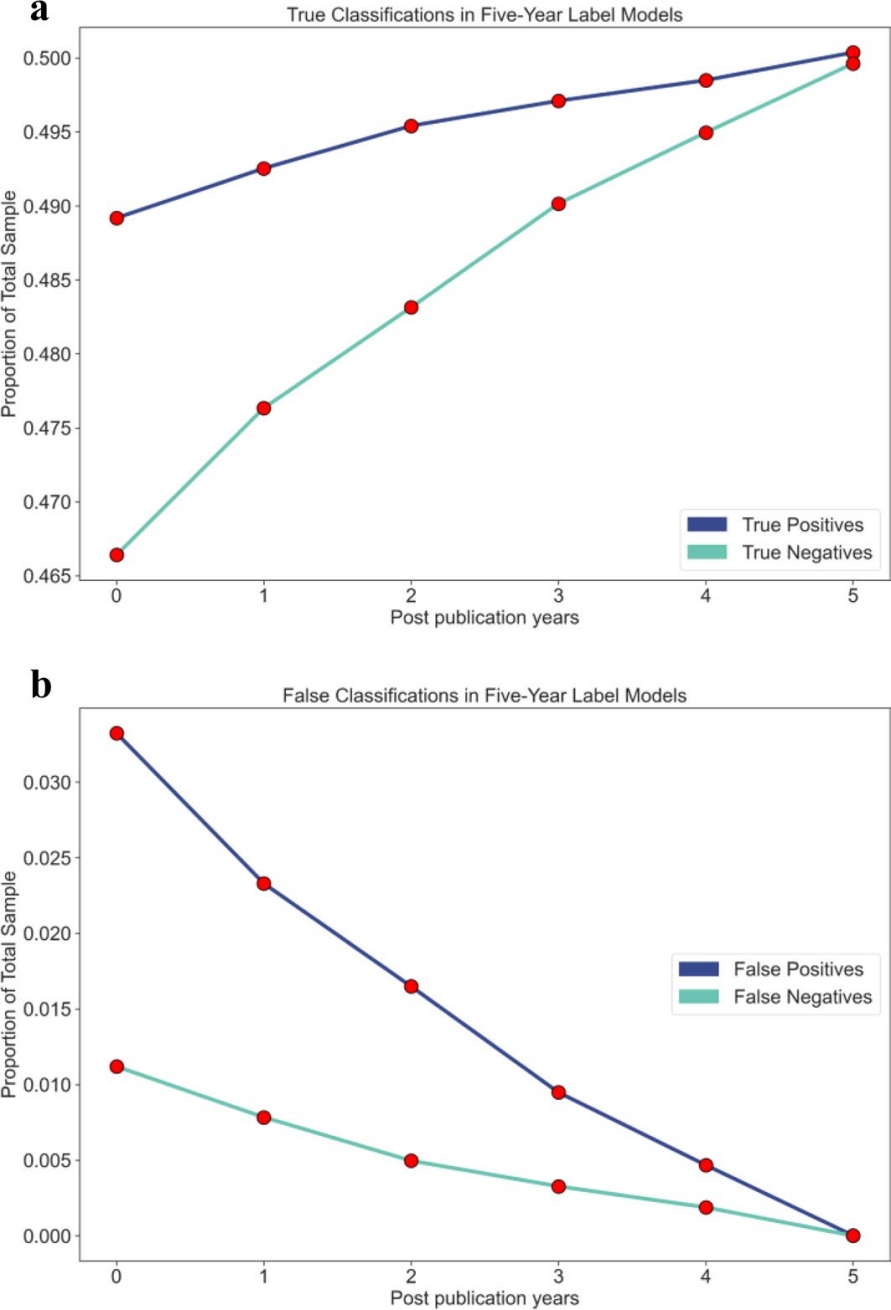
Classification proportions by models. Graphs of classification proportions for trained models with a label five years after publication. Both figures correspond to the models in rows 1–6 of Table 3.

Figure 4 showed that the proportions of false classifications for each model were low. For models with a label five years post publication, the lowest proportion of false positives and false negatives were 0.0332 and 0.012, respectively, for the zero-year model. However, as more data were made available to models, the proportion of false classifications decreased.

The results of our mean decrease in impurity test are detailed in Table 4. The most important variable was rescaled PageRank. This is to be expected as it is the variable used to decide the label. However, its importance is still relatively small at 0.166. The importance of the next four variables sums up to a similar value: 0.158. Notably, these four variables–citations per year, author h-index, maximum citations, and total papers published, are not network variables, but are instead common metrics used to discern the potential impact of a publication. Weighted PageRank and unweighted PageRank, two other network variables, are less important, but still significant, at 0.012 and 0.011 respectively.

**Table 4:** Variable importance.

Variable	Importance value
Rescaled PageRank	0.166
Citations per year	0.049
Author h-index	0.043
Maximum citations	0.042
Total papers	0.024
Citations	0.024
Citations per paper	0.021
Δ Total citations	0.018

The eight most important variables on the 0-year data, 5-year label model, in descending order.

## Discussion

The pursuit of scientific research is intricately tied to the progress of human society, and it is shaped by decisions made by various funding bodies, such as governmental organizations, universities, and both for-profit and nonprofit private entities, who provide financial support for these research studies^1,2^. The National Institutes of Health (NIH), for instance, allocated a budget of 33.34 billion dollars towards scientific research in 2022^18^, highlighting the significant investment made in this area. To ensure the effective utilization of public funds, it is crucial to allocate resources in a way that maximizes favorable outcomes. An analysis done by Fang and Casadevall^19^, observed that biomedical research fund allocation in the United States is inadequate for prioritizing which applications to fund. The present evaluation methodology for impact implicitly relies on quantitative metrics, including the number of publications, citations, and the impact factor of the journals in which researchers publish, as well as related measures such as the h-index. For example, prior work has observed a positive correlation between bibliometrics and NIH funding^20,21^. Furthermore, another study found that NIH-funded researchers had higher h-indices and citation rates than non-funded researchers, and that these differences were more pronounced in certain fields, such as immunology and neuroscience^22^. These articles shed light on some of the potential biases associated with the current bibliometric measures.

There is also growing concern about the potential misuse and abuse of bibliometric measures. In some instances, researchers may engage in self-citation, which artificially inflates their citation counts, or they may publish multiple papers on the same topic to boost their h-index. Thus, relying solely on conventional citation metrics may introduce biases that impede our progress in identifying and pursuing impactful research areas^7,23,24^. In fact, the use of time-rescaled measures of node centrality is an important consideration in knowledge graph analysis for objective decision-making. Its use has already shown promising results in various fields.

According to a study by Kumar et al., the centrality of nodes in a social network can vary over time, making it necessary to use time-rescaled measures of centrality to accurately assess the importance of nodes^25^. A study by Jiang et al. used time-rescaled measures of centrality to analyze the evolution of the Chinese stock market network, identifying key nodes and potential sources of systemic risk^26^. In the context of scientific research, a study by Li et al. demonstrated the effectiveness of using time-rescaled measures of centrality to track the evolution of a knowledge graph in the field of neuroscience, identifying the emergence of new research topics and potential areas for collaboration^27^.

As such we demonstrate the impact of the network framework in predicting high-impact publications. Compared to the models used in Weis and Jacobson, our models showed more favorable performance results when predicting high-impact clinical medicine studies compared to biotechnology studies^16^. In our one- and two-year models for a label year five years post publication, the balanced accuracies are approximately 0.969 and 0.979. On the dataset used in Weis and Jacobson, these numbers were 0.77 and 0.87 respectively.

We observed that the ROC AUC score of each model was greater than 0.99, which indicates that the models have a high level of accuracy in distinguishing between high-impact and low-impact publications (Table 3). The BA scores, which were greater than 0.95, indicate that the models performed well in identifying high- and low-impact cases. Figures 3 and 4 demonstrated that the ROC AUC and BA scores increased with an increase in the amount of data used in the models, except for the three-year post-publication model that predicted impact five years after publication. This is expected since the larger the dataset, the more information was available for the model to learn from; however, the exception observed can be explained by several possible factors including data availability, model design, sample bias, random variation, or noise.

The need for implementation of a time-rescaled measure of node centrality arises from the fact that networks are dynamic and constantly evolving. Using a static measure of node centrality that does not take into account the temporal aspect of the network may not accurately reflect the node's current importance or influence. For instance, a node that was highly central in the network in the past may have lost its importance due to changes in the network, and a node that was less central in the past may have become more important due to new connections or changes in behavior. By using a time-rescaled measure of node centrality, we can better capture the temporal dynamics of the network and get a more accurate representation of each node's current importance or influence^28,29^.

In addition, by representing papers as nodes and their citations as edges, the use of node2vec can learn embeddings that capture the relationships between papers based on their citation patterns. These embeddings can be used to identify clusters of related papers or to detect influential papers that have had a significant impact on the field. By using node2vec to learn embeddings that capture structural and contextual features of nodes in the network, and combining this with time-rescaled measures that reflect the frequency and recency of node involvement in network activity, it is possible to obtain a more nuanced and dynamic understanding of node importance. This approach can help to address some of the limitations of each method when used in isolation, such as the limited interpretability of node2vec embeddings and the sensitivity of time-rescaled measures to hyperparameters and time window size^30^.

The nature of data is often nonlinear, and the implementation of machine learning has led to the discovery of more meaningful outcomes than previously achievable. Machine learning-assisted prediction models have become increasingly prevalent in various scientific fields, particularly in this area of study, and are expected to have a significant impact on decision-making processes. The framework used in this study serves to confirm the reproducibility of the model presented by Weis and Jacobson.

Nonetheless, some limitations arise when using this framework, such as the requirement for historical data, which may not always be available or accessible. In addition, constructing a comprehensive and accurate knowledge graph can be difficult or impossible in some fields, which may limit the applicability of the approach^31^. Another limitation is that the approach may not capture all factors that contribute to impactful research, such as novelty, content, or relevance to current research trends. The knowledge graph structure alone may not always be sufficient to predict the impact of a given paper or research area^32^. Moreover the approach is designed to identify impactful research at the level of individual papers, authors, or research subjects, and may not be directly applicable to other levels of analysis such as journals, conferences, or research communities. Its effectiveness in other contexts may therefore be limited^33^. The model assumes that the knowledge graph is a self-contained representation of the research domain, but impactful research may be influenced by external factors such as funding or policy decisions, which may not be reflected in the knowledge graph^34^. The model may be less effective in domains with complex knowledge graphs or high levels of noise, as it may be difficult to distinguish between significant and insignificant changes in the graph^35^. Future research endeavors should prioritize the prospective validation of our results over time and emphasize the application of our findings specifically to individual research communities or domains.

In the context of defining high-impact papers using a network feature, it is noteworthy to mention that these network features are integral components of the models. This might suggest a potential redundancy, where network features could be perceived as predicting themselves. Nonetheless, forecasting the future state of a network feature, such as the time-rescaled PageRank, is not merely contingent on its current state. Analogously, predicting a child’s future height requires considerations beyond their present height, encompassing factors like nutritional intake and environmental exposures. Thus, the models are enhanced by the incorporation of both network and non-network features. As indicated by Weis and Jacobson, the precise utilization of each variable by the models remains elusive, but insights can be gleaned from the mean decrease in impurity test. It is evident that while time-rescaled PageRank holds significant importance, the subsequent four salient factors are non-network features, cumulatively equating to a comparable importance as the time-rescaled PageRank. This underscores the indispensable nature of both feature types, suggesting that their combined influence fosters a more refined model than either feature type in isolation.

While the efficacy of time-rescaled measures of node centrality has been established, the dynamic intricacies of knowledge graphs necessitate further refinement. The methodologies employed in model construction may not always be optimal. For example, the aggregation of features rooted in author metadata was achieved by averaging said metadata for each publication. A more nuanced aggregation approach, taking into account the author's position in the list, might offer a richer data capture. Moreover, although defining high-impact papers using a feature type present in the model is defensible, a redefined criterion might be more fitting.

Beyond prediction of high-impact studies from bibliometrics, future research is needed to explore the potential to predict high-impact grants that can fund one or more studies. Prospective testing of our study’s methodology applied to research grants can evaluate the extent to which predictive models trained on graph bibliometrics can inform decision-making of funding allocation for multiple studies.

The ramifications of employing time-rescaled measures of node centrality in decision-making warrant thorough assessment. Subsequent studies should delve into the influence of these measures on the precision and efficacy of decision-making, especially in the realm of funding opportunities, and proffer guidelines for their judicious application. Additionally, there is a compelling need for pioneering methods to augment the accuracy of time-rescaled centrality computations. Comparative analyses between these innovative techniques and established ones will be instrumental in discerning their efficacy and relevance.

## Data Availability

The dataset was generated by gathering publication data from Lens Lab, available at https://www.lens.org/. Additional data on publication dates were gathered from Elsevier, available at https://dev.elsevier.com/.

## Code availability

Open-source Jupyter notebooks for model training and evaluation, as well as graph construction and metric extraction, can be found at https://github.com/ldj20/med-predictions.
